# Clinical assessment of computed tomography for detecting ingested blister packs: A single‐center retrospective study

**DOI:** 10.1002/deo2.406

**Published:** 2024-07-15

**Authors:** Yo Ishihara, Chikamasa Ichita, Ryuhei Jinushi, Akiko Sasaki

**Affiliations:** ^1^ Department of Gastroenterology Medicine Center Shonan Kamakura General Hospital Kanagawa Japan; ^2^ Department of Palliative Medicine International University of Health and Welfare Narita Hospital Chiba Japan; ^3^ Department of Health Data Science Yokohama City University Kanagawa Japan; ^4^ Department of Gastroenterology Saitama Medical University International Medical Center Saitama Japan

**Keywords:** drug packaging, eating, endoscopy, foreign bodies, gastrointestinal, tomography, X‐ray computed

## Abstract

**Objectives:**

Blister pack (BP) ingestion poses serious risks, such as gastrointestinal perforation, and accurate localization by computed tomography (CT) is a common practice. However, while it has been reported in vitro that CT visibility varies with the material type of BPs, there have been no reports on this variability in clinical settings. In this study, we investigated the CT detection rates of different BPs in clinical settings.

**Methods:**

This single‐center retrospective study from 2010 to 2022 included patients who underwent endoscopic foreign body removal for BP ingestion. The patients were categorized into two groups for BP components, the polypropylene (PP) and the polyvinyl chloride (PVC)/polyvinylidene chloride (PVDC) groups. The primary outcome was the comparison of CT detection rates between the groups. We also evaluated whether the BPs contained tablets and analyzed their locations.

**Results:**

This study included 61 patients (15 in the PP group and 46 in the PVC/PVDC group). Detection rates were 97.8% for the PVC/PVDC group compared to 53.3% for the PP group, a significant difference (*p* < 0.01). No cases of BPs composed solely of PP were detected by CT. Blister packs were most commonly found in the upper thoracic esophagus.

**Conclusions:**

Even in a clinical setting, the detection rates of PVC and PVDC were higher than that of PP alone. Identifying PP without tablets has proven challenging in clinical. Considering the risk of perforation, these findings suggest that esophagogastroduodenoscopy may be necessary, even if CT detection is negative.

## INTRODUCTION

Blister packs (BPs), also known as press‐through packs, are widely used for tablets and capsules. Cases of BP ingestion account for 28.8%–33.5% of all foreign body ingestions.^1^, ^2^ While complications from foreign body ingestion are rare, occurring in less than 1% of cases, the risk of gastrointestinal perforation increases with sharp or pointed objects like BPs.^3^, ^4^, ^5^, ^6^, ^7^ Gastrointestinal perforations have been documented in several case reports, involving locations including the esophagus,^8^, ^9^, ^10^ small intestinal,^7^, ^10^, ^11^, ^12^, ^13^ and rectum,^10^, ^14^ some of which have been fatal. Therefore, endoscopic removal via gastrointestinal endoscopy is recommended when BPs are ingested.^5^, ^15^, ^16^, ^17^

Because of the risk of perforation due to BP ingestion, early and accurate localization of the object is necessary. While BPs can sometimes appear “UFO”‐shaped on X‐rays,^14^, ^18^, ^19^ their high translucency makes detection by plain radiography challenging.^20^, ^21^ Computed tomography (CT) scans are effective for localizing and diagnosing complications associated with BP ingestion,^9^, ^13^, ^22^, ^23^, ^24^, ^25^, ^26^, ^27^, ^28^ and they can display a triple contrasted target sign consisting of inner high, low, and slightly low‐density layers.^29^ However, the detectability of BPs on CT can vary based on the material; polypropylene (PP) is not detectable, while materials like polyvinyl chloride (PVC) and polyvinylidene chloride (PVDC) are detectable.^21^, ^23^, ^25^ These findings are based on in vitro studies that examined BP alone, rather than actual cases of human ingestion. As no clinical imaging of actual human BP ingestion cases has been conducted, it cannot be conclusively stated whether BPs detectable by CT in vitro are also detectable in clinical settings.

Given that the location, orientation, and presence of tablets within the BPs can significantly affect their detectability in clinical these results may not directly correspond to those observed in vitro. Consequently, this study specifically explored the detectability of different BP materials using CT in actual ingestion cases.

## METHODS

### Ethics consideration

This study was approved by the Institutional Review Board of Mirai Iryo Research Center Inc., Shonan Kamakura General Hospital (Institutional ID: CRB3210004; TGE02133‐024) and was performed in accordance with the principles of the Declaration of Helsinki. Informed consent was presumed unless patients opted out of the study.

### Study design and eligible patients

This retrospective study analyzed cases of accidental ingestion requiring foreign body removal with esophagogastroduodenoscopy (EGD) at Shonan Kamakura General Hospital, Kanagawa, Japan, between 2010 and 2022. The inclusion criteria were accidental ingestion of BP, with the product name and components of the BP being either PP, PVC, or PVDC, and a CT scan performed at the time of diagnosis. The exclusion criteria included cases in which the product name and components of the BP were unknown, a CT scan was not conducted at the time of diagnosis, or the ingested BP was composed of multiple components. We divided the cases into two groups: one comprising cases involving PP (PP group), and the other comprising cases involving either PVC or PVDC (PVC/PVDC group).

### Outcomes

The primary outcome was the CT detection rate. Secondary outcomes were CT value, the incidence of tablet inclusion in the BPs for each group, and the location of BP detection.

### Detection of the BP on CT scan

In this study, cases in which BPs were identifiable on CT were classified as detectable, whereas cases in which BPs could not be discerned on CT were categorized as undetectable. Cases were considered detectable if there were indications in the medical records noted by an emergency physician, and if an experienced internal medicine doctor confirmed the presence of BPs on the CT scan, which was confirmed as a triple contrasted target sign.^29^ Conversely, cases were deemed undetectable if they did not meet these criteria. CT scans were performed using a TOSHIBA Aquilion PRIME system with a tube voltage of 120 kV and a slice thickness of 5.0 mm.

### Definitions of the components in BP

The BP components were identified based on the imprints observed on the packaging in the endoscopic images, which allowed for the determination of the manufacturer and drug name. This information was cross‐referenced with the BP components listed in the medication's package insert. For package inserts that did not mention the BP component, direct inquiries were made to the manufacturers to ascertain the component.

### Statistical analysis

For comparative analysis, the chi‐squared test was used for categorical variables. Continuous variables are presented as medians with ranges or interquartile ranges and were analyzed using the Wilcoxon rank‐sum test. For the primary outcome, the odds ratio and 95% confidence intervals were calculated. R (version 4.2.3, R Foundation for Statistical Computing) was used for statistical analyses. Statistical tests were two‐sided, and *p* < 0.05 was considered statistically significant.

## RESULTS

### Patient characteristics

**FIGURE 1:**
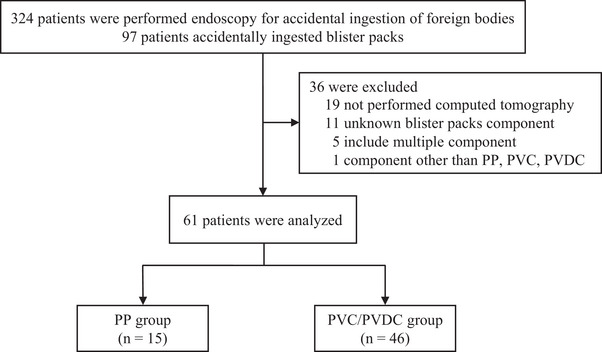
Patient flow. PP, polypropylene; PVC, polyvinyl chloride; PVDC, polyvinylidene chloride.

During 2010–2022, 324 individuals underwent EGD for ingested foreign body removal, of whom 97 (29.9%) had ingested a BP. No patients had gastrointestinal perforation. Although the exact time from ingestion to EGD could not be determined, EGD was performed within 6 h of presentation in all cases. Of these, we excluded 19 patients who did not undergo CT scans, 11 with unknown BP components, five with BPs containing multiple components, and one with a BP component other than PP, PVC, or PVDC. A total of 36 patients were excluded from the study, resulting in a total of 61 patients. The components of the ingested BP were identified as PP in 15 patients (24.6%), PVC in 44 patients (72.1%), and PVDC in two patients (3.3%). They were classified into the PP group (*n* = 15) and the PVC/PVDC group (*n* = 46; Figure 1). The PP group included four men and 11 women, while the PVC/PVDC group included 11 men and 15 women. The median ages were 80 and 74, respectively, with no significant differences observed between the groups (*p* = 0.50). Regarding patients' medical histories, dementia was reported in five patients (33.3%) in the PP group and three patients (6.5%) in the PVC/PVDC group (*p* = 0.026). Ophthalmic disease was reported in one patient (6.7%) in the PP group and none (0.0%) in the PVC/PVDC group (*p* = 0.25). Mental disorder was reported in none (0.0%) of the patients in the PP group and one patient (2.2%) in the PVC/PVDC group (*p* > 0.99). The median number of oral medications was 4 in the PP group and six in the PVC/PVDC group, with no significant difference observed (*p* = 0.35; Table 1).

**TABLE 1:** Characteristics of patients who ingested blister packs.

	Component of blister packages		
	PP group (*n* = 15)	PVC/PVDC group (*n* = 46)	*p*‐value
Male sex, *n* (%)	4 (26.7)	11 (23.9)	1.00
Median age (range)	80 (37, 86)	74 (14, 92)	0.50
History of dementia, *n* (%)	5 (33.3)	3 (6.5)	0.026
History of ophthalmic disease, *n* (%)	1 (6.7)	0 (0.0)	0.25
History of mental disorder, *n* (%)	0 (0.0)	1 (2.2)	1.00
Median number of oral medicines (range)	4 (2, 9)	6 (0, 16)	0.35

Abbreviations: PP, polypropylene; PVC, polyvinyl chloride; PVDC, polyvinylidene chloride.

### Outcomes

The CT detection rates differed significantly between the groups, with 53.3% in the PP group versus 97.8% in the PVC/PVDC group (*p* < 0.01). The CT values for tablets showed no significant difference between the groups, with a median of 129 in the PP group and a median of 134 in the PVC/PVDC group (*p* = 0.97; Table 2). The CT detection rates for cases of BP with tablets were 80.0% in the PP group versus 100% in the PVC/PVDC group, indicating a significant difference (*p* < 0.05). In cases of BPs without tablets, the CT detection rates were 0% in the PP group versus 92.3% in the PVC/PVDC group, also showing a significant difference (*p* < 0.01; Table 3; Figure S1). In the PVC cases, only one BP was undetectable on CT, and it was located in the epiglottis vallecula. The most common location for the detection of ingested BPs was the upper thoracic esophagus, with 29 cases (47.5%), followed by the gastric body with eight cases (13.1%), the middle thoracic esophagus with seven cases (11.5%), and the lower thoracic esophagus with six cases (9.8%). The location information was unavailable and unknown for one case (Table 4). Refer to representative images of CT, EGD, and BPs on PVC, with or without tablets (Figure S2).

**TABLE 2:** Comparison of computed tomography detection rates and values between the polypropylene group and polyvinyl chloride/polyvinylidene chloride group.

Variables	PP group (*n* = 15)	PVC/PVDC group (*n* = 46)	*p*‐value	Odds ratio	Confidential interval
Blister packs with tablet, *n* (%)	10 (66.7)	33 (71.7)	0.96	0.79	0.20–3.54
Detectable on computed tomography scan, *n* (%)	8 (53.3)	45 (97.8)	< 0.01	0.028	0.00056–0.26
Sheet computed tomography value, Hounsfield unit (IQR)	Cannot be detected	152 (128.0, 161.0)			
Tablet computed tomography value, Hounsfield unit (IQR)	129 (59.0, 234.3)	134 (78.5, 180.3)	0.97		

Abbreviations: BP, blister pack; IQR, interquartile range; PP, polypropylene; PVC, polyvinyl chloride; PVDC, polyvinylidene chloride.

**TABLE 3:** Comparison of detection rates of blister packs with or without tablets between the polypropylene (PP) group and polyvinyl chloride/polyvinylidene chloride group.

	PP group	PVC/PVDC group	*p*‐value
Blister packs with tablet, *n*	10	33	
Detectable on computed tomography, *n* (%)	8 (80.0)	33 (100.0)	0.049
Blister packs without tablet, *n*	5	13	
Detectable on computed tomography, *n* (%)	0 (0.0)	12 (92.3)	< 0.01

Abbreviations: PP, polypropylene; PVC, polyvinyl chloride; PVDC, polyvinylidene chloride.

**TABLE 4:** Location of the discovered blister packs.

Location	*n* (%)
Upper thoracic esophagus	29 (47.5)
Gastric body	8 (13.1)
Middle thoracic esophagus	7 (11.5)
Lower thoracic esophagus	6 (9.8)
Antrum of stomach	3 (4.9)
Esophagogastric junction	2 (3.3)
Epiglottis	1 (1.6)
Piriform fossa	1 (1.6)
Cardia of stomach	1 (1.6)
Pyloric antrum	1 (1.6)
Duodenum	1 (1.6)
Unknown	1 (1.6)

## DISCUSSION

In this study involving 61 patients, we found that the CT detection rate was significantly higher in the PVC/PVDC group than in the PP group. The presence of tablets in the BPs also influenced the detection rates, with BPs with tablets showing higher detection rates than those without tablets. Remarkably, the detection rate of BPs without tablets in the PP group was 0%, consistent with that observed in vitro. Nearly half of the ingested BPs were detected in the upper thoracic esophagus.

Prior in vitro studies reported that CT detection of BPs containing PP was challenging, while those containing PVC or PVDC could be detected.^21^, ^22^, ^23^ Another study suggested that BPs might not be detected on a CT scan when the BPs do not include tablets, particularly if no air is trapped within the BPs.^20^ The assessment of ancillary factors, such as air surrounding the tablets, is also essential.^29^, ^30^, ^31^ Our study demonstrated that the CT detection rate for BPs with tablets was higher than for BPs without tablets. Even in cases with BPs containing tablets, the tablets could appear as high absorption findings, enabling the detection of BPs containing PP on CT scans.^23^ Tablets are generally of sufficient size to be extractable in standard 5 mm CT slices, making them much easier to identify than thin BPs. In addition, a previous study reported that ingested BPs are most commonly found in the esophagus, with a prevalence ranging from 79–90.9%.^32^, ^33^ Notably, ingested BPs are frequently discovered at the first natural constriction in the upper thoracic esophagus,^2^, ^34^ which is consistent with our study in which most ingested BPs were detected in the upper thoracic esophagus; therefore, focusing the search on the esophagus may be important to detect BPs on both CT scan and EGD.

The higher CT detection rates of PVC/PVDC BPs were influenced by the presence of chloride atoms in the material. Within the composition of BPs, chlorine atoms, which have the highest atomic number among the constituents, contribute to an increased electron density, leading to higher CT values for PVC and PVDC.^21^ In contrast, PP, which does not contain chlorine atoms, does not have a high CT value; thus, PP could not be detected on the CT scan. This underscores the relationship between the material composition of BPs and their detectability on CT. Incidentally, in one case, a PVC BP without tablets, located in the epiglottic vallecula, was completely undetectable on axial CT (Figure S3). This may have been due to not being captured in the 5mm axial slices. Evaluating CT images in sagittal and coronal views might aid in the detection of BP.

This study, examining CT detection rates by material type in actual cases of BP ingestion, makes a unique contribution to the research on this topic. The results of this study contribute to the diagnostic and endoscopic treatment decisions for BP ingestion that clinicians often encounter. Specifically, it highlights that, in cases where BP ingestion is suspected based on the patient's history, even if it is not detected on CT, EGD focused on observing the esophagus is necessary. Furthermore, by identifying ingested BPs, it is possible to reasonably predict whether BPs are detectable using CT before testing.

This study had several limitations. First, it was constrained by its single‐center nature. In addition, the small sample size limited our ability to thoroughly examine all potential confounding factors. Being a retrospective study is also a limitation. Future research should focus on collecting larger datasets to better ascertain the applicability of our findings in clinical practice.

In conclusion, the CT detection rate of BPs ingested by humans varies depending on their components, with PVC and PVDC showing higher detection rates than PP. Detecting BPs composed of PP without tablets via CT scan is challenging. Therefore, when BP ingestion is suspected based on patient history or symptoms and considering the risk of gastrointestinal perforation, EGD is recommended for retrieving BPs.

## CONFLICT OF INTEREST STATEMENT

None.

## ETHICS STATEMENT

This study was approved by the Institutional Review Board of Mirai Iryo Research Center Inc., Shonan Kamakura General Hospital (institutional ID: CRB3210004; TGE02133‐024) and was performed in accordance with the principles of the Declaration of Helsinki.

## PATIENT CONSENT STATEMENT

Informed consent was presumed unless patients opted out of the study.

## Supporting information


**Figure S1** Summary image showing computed tomography detection rates in blister packs with and without tablets.
**Figure S2** Representative images of computed tomography, esophagogastroduodenoscopy, and blister pack on polyvinyl chloride with tablet (a–c), and without (d–f). Blister pack is indicated with a white arrowhead on the computed tomography scan.
**Figure S3**. Ingested case of blister pack of polyvinyl chloride without tablet. Blister pack was unable to detect on computed tomography (a). Blister pack was detected in the epiglottic vallecula and removed by esophagogastroduodenoscopy (b, c).
