# Supplementary figures and images for: Clinical assessment of computed tomography for detecting ingested blister packs: A single‐center retrospective study
Source: DEN Open. 2024 Jul 15;5(1):e406. doi: 10.1002/deo2.406 (PMC11248714; doi:10.1002/deo2.406)

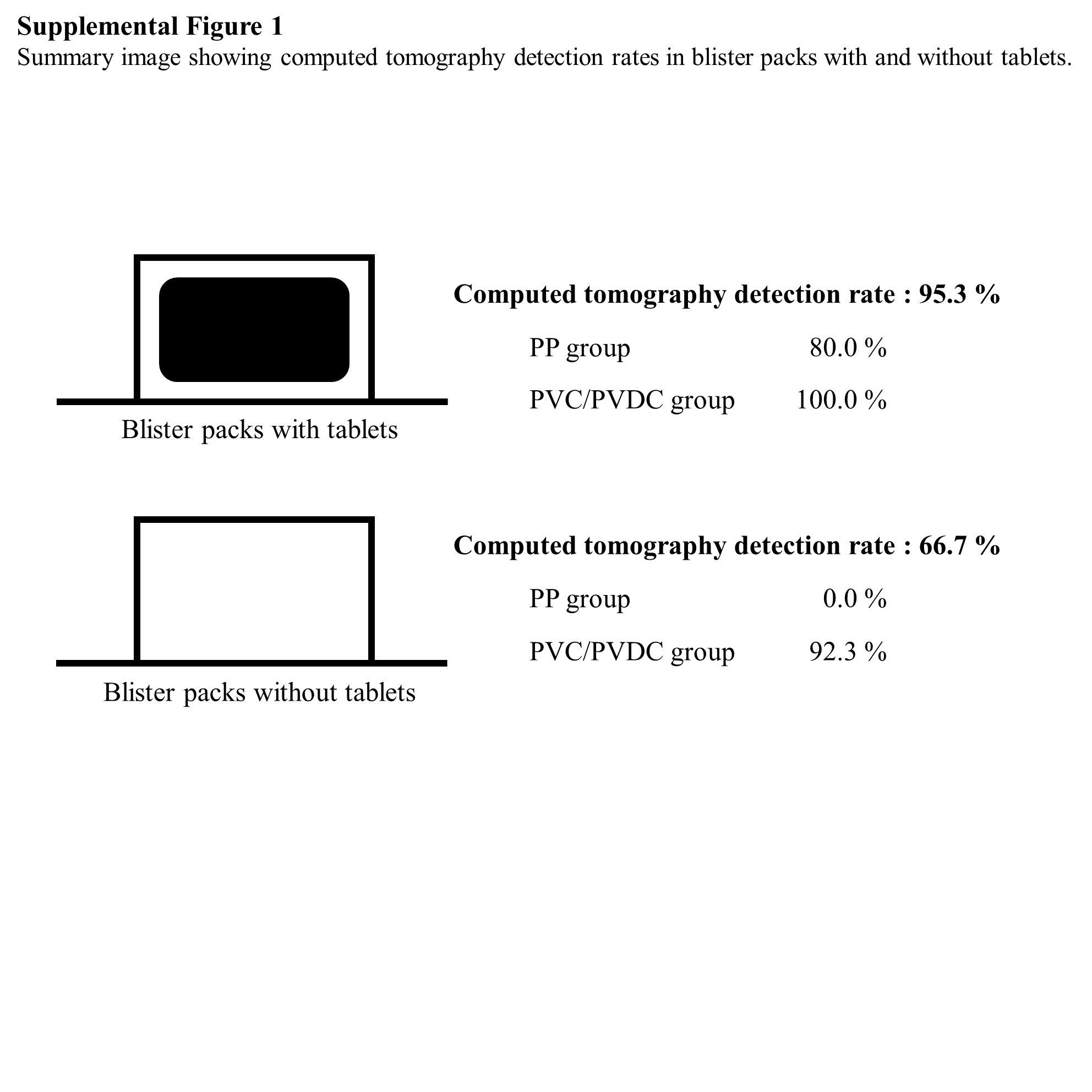

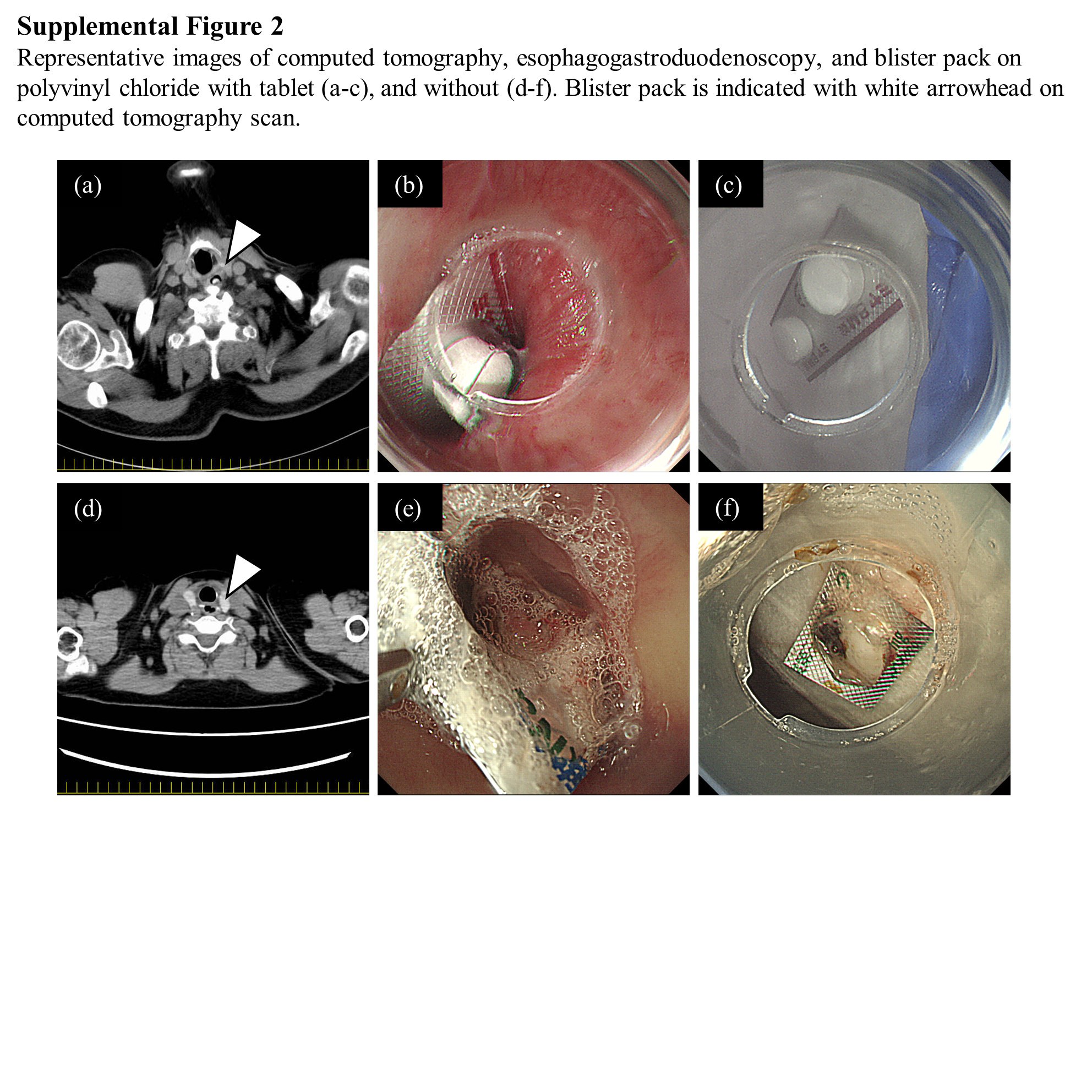

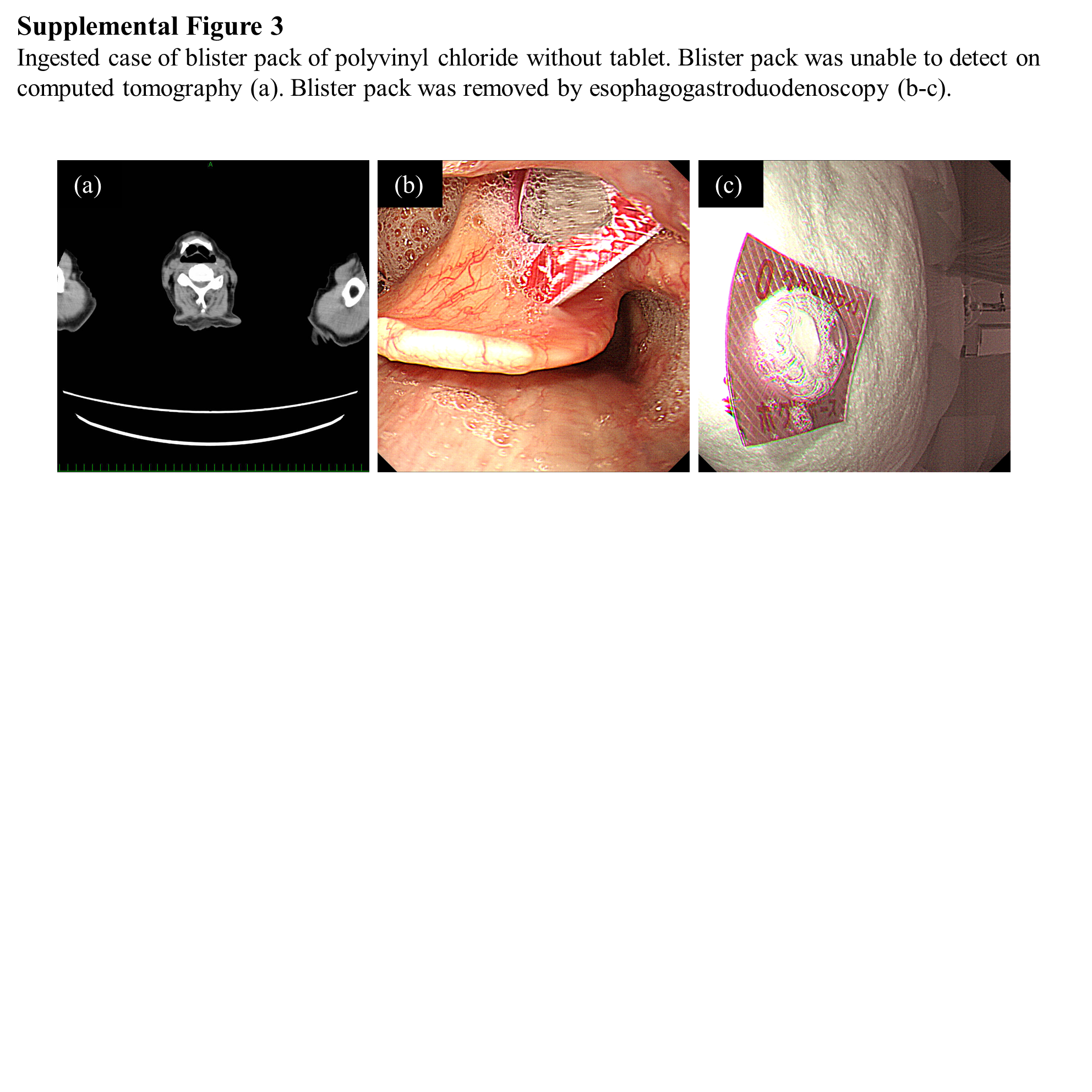

Supplement: Supplementary file 1 — Figure S1 Summary image showing computed tomography detection rates in blister packs with and without tablets. Figure S2 Representative images of computed tomography, esophagogastroduodenoscopy, and blister pack on polyvinyl chloride with tablet (a–c), and without (d–f). Blister pack is indicated with a white arrowhead on the computed tomography scan. Figure S3. Ingested case of blister pack of polyvinyl chloride without tablet. Blister pack was unable to detect on computed tomography (a). Blister pack was detected in the epiglottic vallecula and removed by esophagogastroduodenoscopy (b, c). [file DEO2-5-e406-s001.docx]
